# When stories become care: mapping literary exposure to empathy and resilience in medical students and doctors through the lens of narrative medicine

**DOI:** 10.3389/fmed.2026.1789725

**Published:** 2026-05-08

**Authors:** S. Jennifer Sandra, Daisy Gohain

**Affiliations:** School of Social Sciences and Languages, Vellore Institute of Technology, Chennai, Tamil Nadu, India

**Keywords:** empathy, iatrogenic suffering, literary exposure, medical humanities, narrative medicine, resilience

## Abstract

**Introduction:**

This study explores the relationship between literary exposure and characteristic traits such as empathy and resilience. It examines this association as a potential factor associated with the development of clinical judgement in clinical care. This examination contributes to understanding factors related to “iatrogenic suffering” of the patients. Using Narrative Medicine as the conceptual framework, the study adopted a quantitative methodology by surveying medical students and doctors to explore how their engagement with literature, films, and other narratives relates to their orientation toward patient-centered care.

**Methods:**

A structured online questionnaire consisting of five sections including: Demographics, Literary Exposure, Perception of Literary Value, Empathy, and Emotional Resilience, was circulated among medical students and doctors across Tamil Nadu, India. Using purposive and convenience sampling, 120 responses were collected with informed consent. The data were analyzed using SPSS and Python software. The research employs Pearson and Spearman’s correlation along with Simple Linear Regression Analysis to explore the relationship between exposure to the Humanities, especially literature, and Medicine, in order to determine its association with certain characteristic traits such as empathy and resilience.

**Results:**

The findings indicate a statistically significant association between consistent literary engagement and higher self-reported empathy and resilience. This quality has been linked in prior scholarship to greater sensitivity to doctor-induced patient suffering.

**Discussion:**

Thus, literary and narrative engagement is associated with an orientation toward compassionate care. On this basis, this study suggests that literary narratives may be considered a meaningful component within medical education, while acknowledging the correlational nature of these findings.

## 1 Introduction

In today’s medical world, doctor-patient consultation can work in ways that either ease or increase the pain and suffering of the patients. This becomes significant especially in the sensitive areas such as palliative care, where the responsibility of the doctor extends beyond clinical treatment to addressing the emotional realities of illness. The advances in medical technology have equipped the doctors to diagnose and treat life-threatening diseases like cancer. However, there is a growing concern that surrounds reduced attention to emotions, narrative comprehension, and relational sensitivity (1, 2). In fast-paced medical environments, many patients feel unseen or insufficiently valued, particularly when treatment decisions precede the lived experience of illness and the existential questions that accompany it.

There are multiple systemic and situational factors that affect and influence the doctor-patient interactions. Time constraints, work-pressure and the demands of clinical efficiency limit the space available for meaningful engagement. As a result, physicians may unintentionally contribute to patient distress through emotionally detached or depersonalized communication (3, 4). Kuhl (5) conceptualizes this phenomenon as “iatrogenic suffering” referring to the inadvertent harm caused by the absence of empathetic and attentive communication and not by medical error. This was further highlighted by Whitehead (6) in his research where he talks about the lived experiences of the physicians who deal with patient death. This concern is specifically relevant in palliative care, where patients often seek validation and acknowledgment of their subjective experiences along with the medical intervention. Despite extensive training in medical protocols and treatment procedures, physicians may not always be equipped to interpret and respond to the complexities of human pain, suffering, fear, and hope. This calls for a reimagination of how emotional and ethical abilities are developed within medical training. These concerns have led to the growing interest in approaches that foreground the human dimensions of medical practice.

This study is situated at the intersection of medical humanities, narratology, and medical education, contributing to the ongoing interdisciplinary discussions in patient-centered care. Scholars have increasingly argued for a shift in medicine from a purely technical and scientific domain toward a more humanistic practice that recognizes the patient as a living person rather than a clinical case. Within this context, the integration of humanities into medical education has been proposed as one way of addressing the concerns about declining empathy in clinical practice (7). Engagement with literature, storytelling, and reflective practices provides opportunities for medical students to encounter diverse human experiences, fostering ethical sensitivity and emotional awareness.

Narrative medicine, developed by Charon (8), has gained popularity as a pedagogical framework that emphasizes the importance of storytelling in clinical care. It encourages physicians to pay attention to patients’ narratives and to cultivate “narrative competence,” which is the ability to recognize, interpret, and respond to stories of illness. In this regard, literature encompasses complex emotions, ambiguity and subjective accounts of experience. These are the dimensions that are usually underrepresented in conventional medical training. This has been described as a compensatory tool for dominant medical pedagogy that focuses on objectivity and technical training, as it enables medical students to engage with delicate situations and uncomfortable conversations (9, 10). Nevertheless, the medical curriculum continues to undervalue the potential of literature.

Existing research in the field of narrative medicine and medical humanities has explored the relationship between narrative engagement and psychosocial traits such as empathy and resilience. Studies suggest that exposure to patient stories, memoirs, and reflective literature is associated with enhanced empathetic understanding and improved capacity to engage with patient suffering (11, 12). A considerable number of qualitative studies have further demonstrated that literary engagement can strengthen communication skills, foster empathy, and support coping with emotional exhaustion, thereby contributing to resilience among medical practitioners (13, 14). Several experimental and quasi-experimental studies involving narrative-based interventions in medical education have also reported improvements in empathy and humanistic orientation (10, 12, 15).

Recent scholarship has extended these insights by examining the role of narrative medicine in structured and longitudinal educational contexts. For instance, Huang et al. (16) highlight its contribution to holistic care by fostering the psychosocial awareness and ethical sensitivity. Similarly, Paul et al. (17) demonstrate its relevance in palliative care training, particularly in enhancing reflective practice and resilience. Contemporary scoping reviews in medical education (18) further indicate that narrative pedagogy supports professional identity formation and patient centered communication across clinical contexts.

Indian scholarship provides an important regional perspective on these developments. Bhargava et al. (19) document the feasibility of integrating storytelling and narrative reflection into community medicine training, while Prabhu (20) emphasizes the role of literature in cultivating ethical sensitivity and holistic awareness in medical classrooms. In the context of palliative care, Haridas (21), reflects on the end-of-life care through a narrative lens, highlighting the importance of attending to patients’ lived realities. While these studies emphasize the value of narrative engagement, there is a lack of empirical research regarding the extent to which such exposure translates into measurable clinical competencies across diverse contexts. The evidence base is uneven in its methodological and contextual scope.

Despite increasing attention to narrative -based pedagogical frameworks (3, 22), medical education continues to prioritize clinical knowledge, technical skills, and quantifiable outcomes over emotional and narrative competencies. This institutional emphasis marginalizes literature as a non-essential and “soft discipline,” limiting its integration into formal curricula and research frameworks. However, humanities-based approaches, particularly literature, have supported the development of ethical sensitivity and emotional awareness in clinicians (2, 9). Although narrative medicine is widely discussed, its effect on specific psychosocial traits is not supported by empirical data. As a result, there remains a gap in systematically examining how literary engagement corresponds with measurable dimensions such as empathy and resilience.

Addressing this gap, the present study explores the relationship between literary exposure and selected psychosocial traits among medical students and practicing doctors. By examining the patterns of engagement with literature and their association with empathy and resilience, the study seeks to contribute empirical support to ongoing discussions in medical humanities. In doing so, it positions literary engagement as a meaningful component within medical education instead of placing it as a cultural or recreational activity.

## 2 Objectives and hypotheses

The primary objective of this study is to investigate how varying levels of engagement with literary and other forms of narratives correspond with self-reported empathy and resilience among medical students and professionals. With narrative medicine as the conceptual foundation, this research aims to examine the association between literary exposure and the development of psychosocial traits such as empathy and resilience. Both of these qualities are essential for compassionate and patient-centered care.

In order to achieve this objective, two main hypotheses were formulated, each pertaining to one psychosocial trait. These were evaluated using Correlation and Simple Linear Regression analysis using the data collected from 120 respondents from Tamil Nadu, India. The respondents include medical students and practicing doctors. The hypotheses are as follows:

*H01*: There is no significant relationship between literary exposure and empathy among medical students and practitioners.

*H11*: There is a significant positive relationship between literary exposure and empathy among medical students and practitioners.

*H02*: There is no significant relationship between literary exposure and resilience among medical students and practitioners.

*H12*: There is a significant positive relationship between literary exposure and resilience among medical students and practitioners.

## 3 Conceptual foundation

This exploratory study employs narrative medicine (8) as a conceptual and pedagogical framework as it situates the practice of medicine within the world of storytelling. Developed by Charon (8), this approach contends that the clinical conversation between a doctor and a patient does not just involve diagnosis and medication but it is a narrative exchange where the patient’s story, with all the lived experiences, is central to understanding illness. Charon introduces a concept called the “Narrative Competence,” which is the “ability to recognize, interpret and respond to the stories of others” (8). It can be viewed as an essential skill that develops empathy, resilience and ethical awareness in clinical care. Medical students who are exposed to literary texts and other narratives are also exposed to different aspects of human life such as suffering, pain, fear, hope and happiness.

Narrative Medicine as a medical practice is valued in palliative care. Terminally ill patients expect the doctors to listen to them, not just to prescribe medicines. This framework enables clinicians to understand and identify ways in which their language, tone and gesture can make the patient feel valued, comforted, and respected. When the doctors cultivate empathy and resilience, they are better equipped to reduce “iatrogenic suffering” (5), which is the inadvertent harm caused by insensitive or emotionally detached communication. Thus, narrative medicine provides a conceptual lens for understanding how literary engagement fine-tunes the clinical judgment and is associated with the development of empathy and resilience.

In this study, Narrative Medicine functions as a conceptual lens and pedagogical stance. It offers a way to understand literary engagement in relation to medical students’ and doctors’ empathy and resilience, which are central to patient-care. Here, stories become a tool, and the framework reiterates the idea that the Humanities plays an integral role in the moral and emotional education of doctors.

## 4 Materials and methods

This study employed a quantitative methodology using correlation and linear regression to examine the relationship between literary exposure, empathy and emotional resilience among medical students and doctors in Tamil Nadu, India. It sought to explore how medical students and practitioners who are consistently exposed to literary narratives are linked to empathy and emotional resilience in a better way. These two attributes are the core psychosocial traits that pave the way to a humane clinical practice. A quantitative method was chosen since it allows for the measurement of the relationship between the variables. Given the cross-sectional design of the study, causal inferences cannot be drawn. Rather, the results indicate meaningful associations between literary exposure and self-reported levels of empathy and resilience.

Data for this study was collected through an online survey using Google form. A purposive and convenience-based sampling was carried out, ensuring that the participants of the survey represented medical students and practicing doctors. However, this approach does not yield a sample that is statistically representative of the broader population of medical students or healthcare professionals. Therefore, the findings should be interpreted as context-specific and indicative rather than generalizable. The questionnaire was shared online with medical students and practicing doctors across Tamil Nadu, India. A total of 120 responses were collected. Every participant was clearly informed about the purpose of the study and they gave their consent before accessing the Google form. All the responses were kept anonymous to maintain privacy and confidentiality.

The form had five main sections: Demographics, Literary exposure, Perception of literary value, Empathy, and Emotional resilience. Literary exposure was measured by combining scores related to how often participants engaged with literature and the kind of literary or narrative forms they interacted with. Empathy and resilience were tested using adapted and context-specific items informed by established instruments, such as Jefferson’s Scale of Empathy (23) and Connor-Davidson Scale of Resilience (24). The items were modified to align with the clinical and educational context of medical students and practitioners, ensuring conceptual relevance while not constituting a direct replication of the original scales. The original instruments were used solely as scholarly references for non-commercial academic purposes and are appropriately cited.

During this process, the core conceptual domains associated with empathy and resilience were taken as a reference while developing context-specific items for the study. Items were phrased and contextualized to align with narrative engagement and clinical experience (Table 1). Particularly, the items related to perspective-taking, emotional understanding, and reflective practices informed the construction of the items 11–15. The resilience-related items that focussed on emotional regulation, recovery and meaning-making informed items 16–20. Additionally, a set of items (1–10) were developed to operationalize Literary exposure Score (LES), capturing both frequency of engagement (Items 1–5) and perceived impact of narratives (Items 6–10).

**TABLE 1:** Questionnaire items.

S. No.	Section	Questionnaire items
1.	B	Read fiction (novels, short stories, plays).
2.	B	Watch films that are extremely emotional.
3.	B	Read or watch real life patient stories, memoirs or autobiographies (films/books that share the stories of the patients).
4.	B	Engage with stories or media (films, articles, real life stories, news, books, social media) that involve human suffering, healing, or caregiving.
5.	B	Watch medical dramas and web series that are available on the various OTT platforms.
6.	C	Stories help me understand patient perspectives.
7.	C	Watching films or reading stories develops my empathy.
8.	C	Literature/narratives help me to reflect on ethical dilemmas in medicine.
9.	C	Exposure to different stories influences how I interact with patients.
10.	C	Narratives/stories help me to communicate with others in a better way.
11.	D	I will try to imagine myself in my patients’ shoes.
12.	D	Understanding body language of the patients is as important as verbal communication in patient care.
13.	D	Empathy is a very important factor in a doctor-patient relationship.
14.	D	Patients feel valued and comfortable when doctors understand their feelings.
15.	D	I often think about or reflect on patient conversations/stories about patients.
16.	E	I stay calm under emotional pressure.
17.	E	I try to recover quickly after an emotionally deep conversation.
18.	E	I try to find meaning/resolution to the challenges I face during medical practice.
19.	E	I use creative/artistic expression (e.g., writing, reading, watching films) to manage stress.
20.	E	I can empathize with others without being overwhelmed.

These additions were designed to extend the underlying constructs and not to reproduce the original instruments. The adapted instrument was reviewed for content clarity and contextual relevance, and its internal consistency reliability was accessed using Cronbach’s alpha. The results indicated acceptable to good reliability across the scales. However, factor analysis and other formal construct validation procedures were beyond the scope of this exploratory study. This is acknowledged as a limitation, with scope for further validation in future research.

The internal consistency reliability of the study instruments was calculated using Cronbach’s Alpha within the present sample (*N* = 120). The Literary Exposure Scale (10 items) demonstrated good internal consistency (α = 0.84), indicating strong coherence among the items measuring literary engagement. The Empathy Scale (5 items) yielded acceptable reliability (α = 0.78), suggesting a satisfactory internal consistency following contextual adaptation for the present study. The Resilience Scale (5 items) produced a reliability coefficient of (α = 0.65), reflecting moderate internal consistency. Given the limited number of items and the multidimensional nature of resilience, this value was considered adequate for exploratory analysis in the current interdisciplinary research context.

Statistical data analysis software such as SPSS was used to ensure credibility and accuracy. Firstly, correlational analyses were carried out to examine the associations between literary exposure and empathy, and between literary exposure and emotional resilience. Pearson’s Correlation Coefficient and Spearman’s rho were employed for normally distributed data and ordinal variations respectively. Literary exposure was subsequently tested using linear regression analysis to assess its association with empathy and emotional resilience. The combination of both correlation and regression analyses enabled the identification and characterization of relationships between the variables within the sample. This approach provides a comprehensive view of the association of literary exposure with the core psychosocial traits, in this case, empathy and emotional resilience.

Given the exploratory and cross-sectional nature of the study, the analysis was intentionally limited to bivariate and regression techniques to establish baseline relationships. The aim of the research was to identify and empirically quantify observable patterns that may inform future multivariate research and not to construct a predictive model. The characteristics focussed in this study are generally shaped by individual experiences and not by controlled experimental conditions. The correlation and regression analyses helped bring out the association between the variables. This ensured the accuracy and reliability of the statistical calculations. This analysis aimed to examine the association between literary exposure, empathy, and resilience. Therefore, the study contributes empirical support to discussions at the intersection of medical education and the humanities by showing that literary engagement is associated with the psychosocial traits examined here.

All ethical guidelines for research involving human participants were carefully followed and approval for the same was obtained from the Institutional Ethical Committee for Studies on Human Subjects. Participation was completely voluntary, and every participant gave their informed consent before accessing the survey. They were clearly informed about the aim of the study, data privacy and that they could withdraw at any time. No personal details were collected, and all responses were securely stored and used only for academic purposes.

## 5 Findings and discussion

### 5.1 An overview of the variables

**FIGURE 1:**
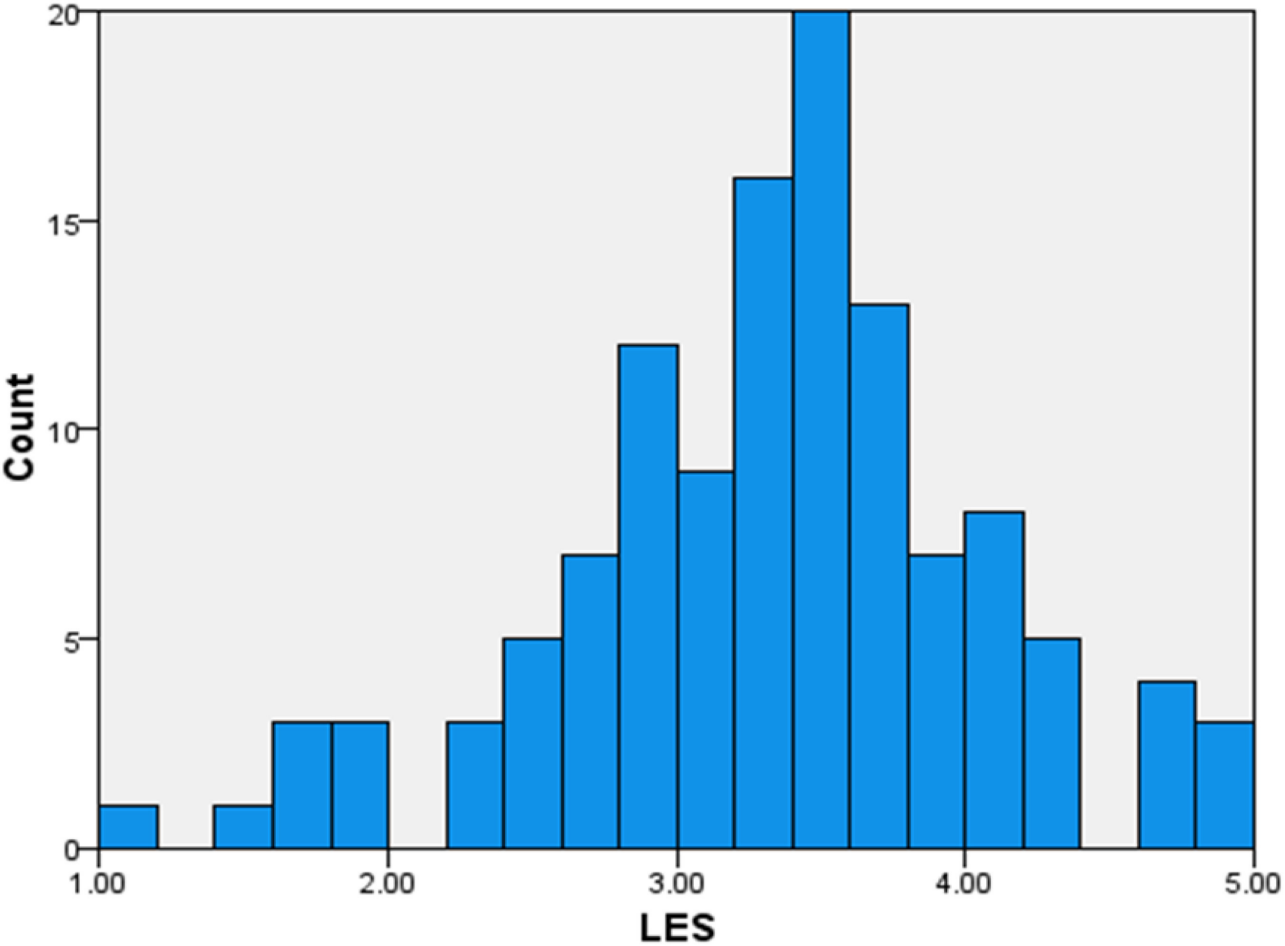
A visual representation of literary exposure score (LES).

A total of 120 responses were collected from medical students and practicing doctors across Tamil Nadu. Each participant was tested using three main variables including: Literary Exposure Score (LES), Empathy Score (ES) and Resilience Score (RS). The LES (Figure 1) reflected how often participants engaged with different narrative forms such as novels, short stories, films and series. The ES indicated how well they could understand their patients’ emotions and RS represented their ability to recover from stressful experiences in the medical settings.

**FIGURE 2:**
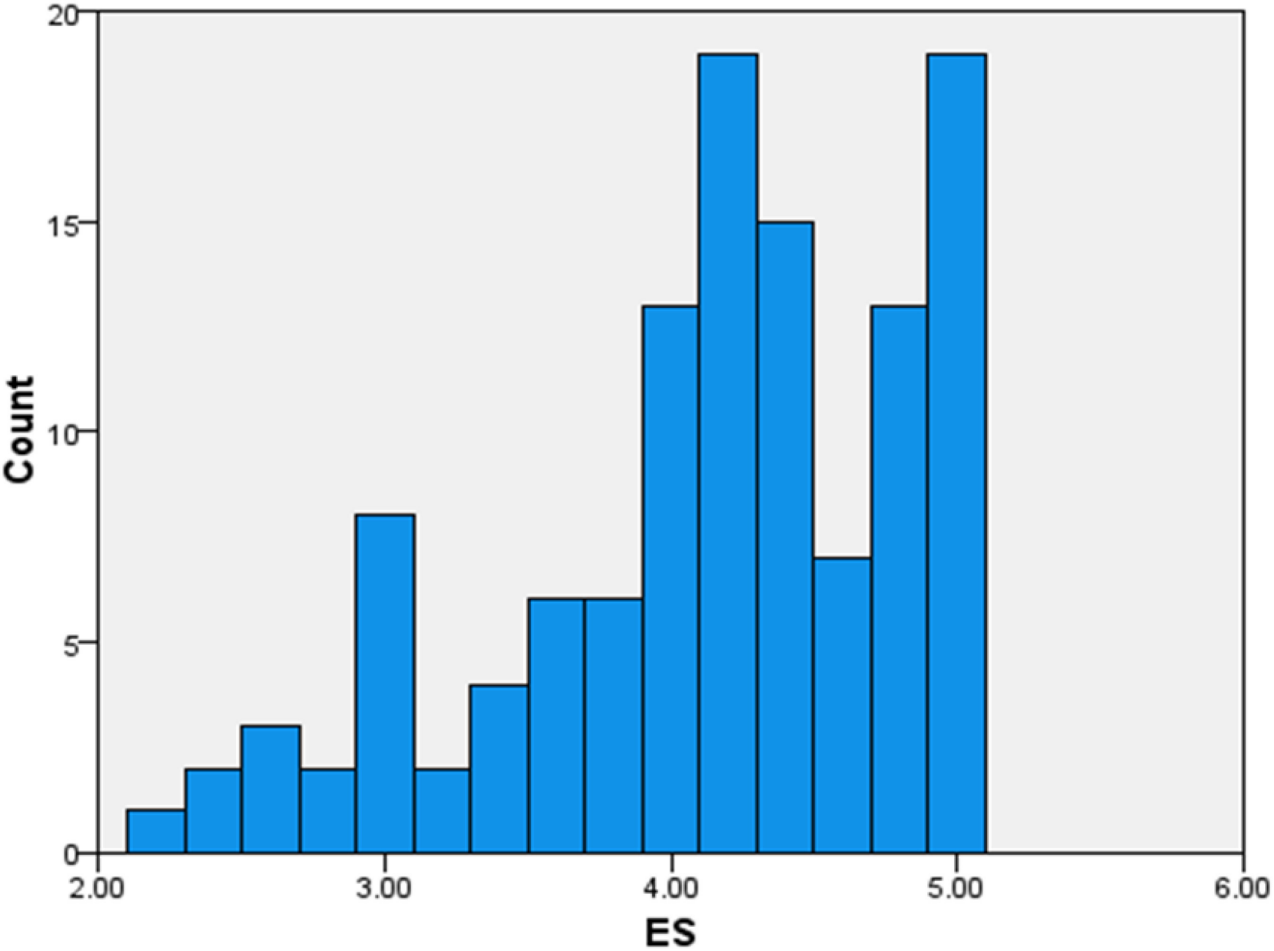
A visual representation of empathy score (ES).

The overall scores were mostly normal, with only slight differences between the subgroups. The Literary Exposure Score ranged from moderate to high, showing that most participants were well engaged with literature and other narrative forms. The Empathy Scores (Figure 2) were also similar to LES, recommending that the participants identified empathy as an essential quality in their medical practice.

The current study employs one-tailed significance testing based on the a priori directional hypotheses derived from both Narrative Medicine and Medical Humanities literature, which consistently suggest a positive association between literary engagement and psychosocial constructs such as empathy and resilience. The hypotheses (H11 and H12) deliberately predicted a positive relationship rather than a non-directional association and so one-tailed tests were considered appropriate for exploring the relationship in the specific direction. However, it is acknowledged that two-tailed tests are more commonly reported in empirical research as a conservative standard. Notably, the observed relationships remain statistically significant even under two-tailed testing (*p* < 0.001). This indicates that the choice of one-tailed testing does not alter the substantive interpretation of the findings.

### 5.2 Correlation between literary exposure and empathy

**FIGURE 3:**
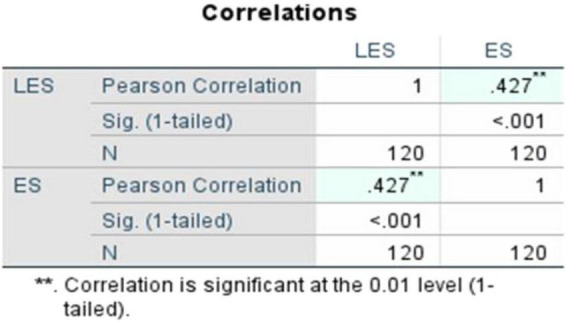
Pearson’s correlation between literary exposure score and empathy score.

**FIGURE 4:**
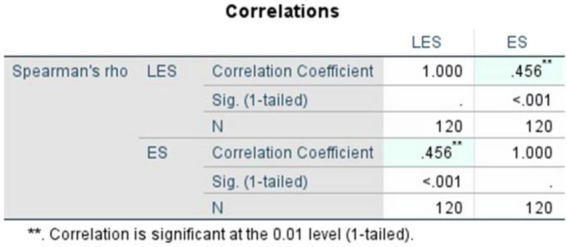
Spearman’s rho (literary exposure score and empathy score).

To statistically investigate this relationship, Pearson’s correlation coefficient was calculated as the primary measure and Spearman’s rho was used to check the credibility of the tests. Both the correlational analyses indicated a statistically significant, and moderately positive correlation between LES and ES. The Pearson’s correlation coefficient (Figure 3) is reasonably moderate, recorded at *r* = 0.427 and its significance (1-tailed, *p*) = < 0.001, demonstrating that the relationship is statistically significant and moderately strong. Likewise, to ensure the robustness of this finding, Spearman’s rho (Figure 4) was calculated and recorded at ρ = 0.456 with its statistical significance (1-tailed, *p*) = < 0.001. This supports the consistency of the observed relationship across methods. These findings reiterate the positive linear association between both the variables LES and ES.

These findings empirically support Narrative Medicine’s proposition that sustained engagement with stories is associated with the capacity to understand, interpret and respond to the emotional states of the others. Medical practitioners who are consistently exposed to literature and other forms of narratives such as films and series encounter emotionally complex characters. This engagement with narratives can be seen as forms of mediated experiential learning. Here, repeated engagement appears to correlate with their ability to identify and recognize similar emotional nuances, and to sensitively listen to the patient narratives. Empathy is not just a descriptive construct but a cognitive and interpretative capacity that may develop through sustained narrative engagement. In this sense, the observed correlation acts as quantitative indicators of a meaningful association between narrative exposure and empathetic orientation in clinical settings and not as proof for direct transformation.

Furthermore, this finding echoes Charon’s (25) argument that narrative practice enables doctors to connect emotionally to the patients without disturbing the professional distance between them. This study does not position narrative engagement as a deterministic tool, but the association between LES and ES suggests that it may function as a pedagogical resource within the broader web of factors that shape empathy. This is relevant within patient-centered care, where empathy functions as a contextually developed skill that is influenced by other factors.

### 5.3 Correlation between literary exposure and resilience

The next part of the analysis looked at how the Literary Exposure Score (LES) is related to the Resilience Score (RS). In simple terms, resilience in medicine refers to the ability to recover after emotionally difficult situations. Here, resilience plays an important role as it protects the doctor’s own wellbeing and ensures that patients receive compassionate care. When resilience is low, doctors are more likely to experience burnout and emotional exhaustion. This will in turn affect their performance and their connection with the patients.

**FIGURE 5:**
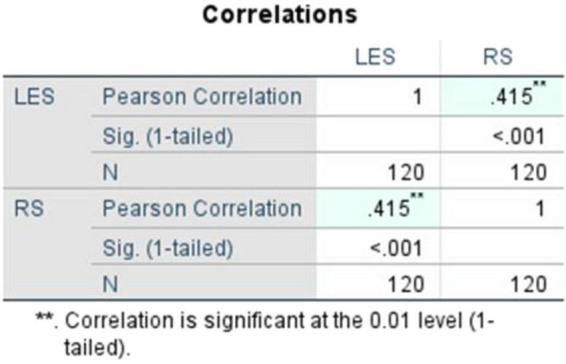
Pearson’s correlation between literary exposure score and resilience score.

**FIGURE 6:**
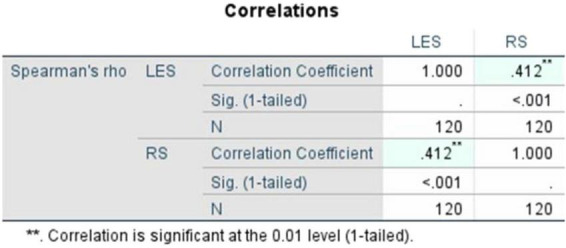
Spearman’s rho (literary exposure score and resilience score).

The correlation findings showed a positive linear association between literary exposure and resilience. Pearson’s correlation coefficient (Figure 5), recorded at r = 0.415 and its significance (1-tailed, *p*) = < 0.001, demonstrates that the relationship is statistically significant and moderately strong. Likewise, to verify the soundness of the observed finding, Spearman’s rho (Figure 6) was computed and recorded at ρ = 0.412, with its statistical significance (1-tailed, *p*) = < 0.001. This ensures the consistency of the association. These findings emphasize the moderately positive, and statistically significant linear association between both the variables LES and RS.

These findings advocate for the concept that engaging with stories may help doctors and medical students to process their experiences reflectively instead of internalizing them without articulation. As Charon (8) and Stoffel and Cain (4) suggest, narrative practices encourage structured reflection, giving them a way to interpret and reorganize difficult moments in a meaningful way. Liao (1) describes this as a “structured empathy practice”. It is an interpretative space where doctors can explore emotional complexity without fear of clinical consequences. This space may function as a form of cognitive and emotional rehearsal, allowing the doctors to explore responses to suffering in a reflective manner. Exposure to stories and films doesn’t just build empathy, it also appears to be associated with the development of emotional regulation and interpretative flexibility (11, 13).

The results of this study suggest a meaningful relationship between sustained exposure to literary narratives and the resilience of the doctors. Here, literary exposure appears to align with emotional resilience, as a part of a broader process of reflective development and not as a direct outcome. This may enable doctors to recognize and respond to suffering through understanding rather than detachment. As noted by Chye et al. (13) and Shorbagi et al. (22), resilience is not a fixed trait but a quality that can be developed over time through practice. From this perspective, Humanities offers practical tools that may aid in the development of such strength. The moderately positive and statistically significant correlation found here provides empirical support for this association, yet, suggesting that such a development occurs within a wider network of personal and other influences.

### 5.4 Regression analysis

Using the Literary Exposure Score (LES), Empathy Score (ES) and Resilience Score (RS), simple linear regression analyses were conducted to further examine the observed relationships. In this study, regression was used to assess the association between literary exposure and the psychosocial traits under consideration, within the sample. Instead of operating as a predictive model, here, the regression analysis was used to complement the correlational findings by describing the direction and strength of these associations.

#### 5.4.1 LES and ES

**FIGURE 7:**
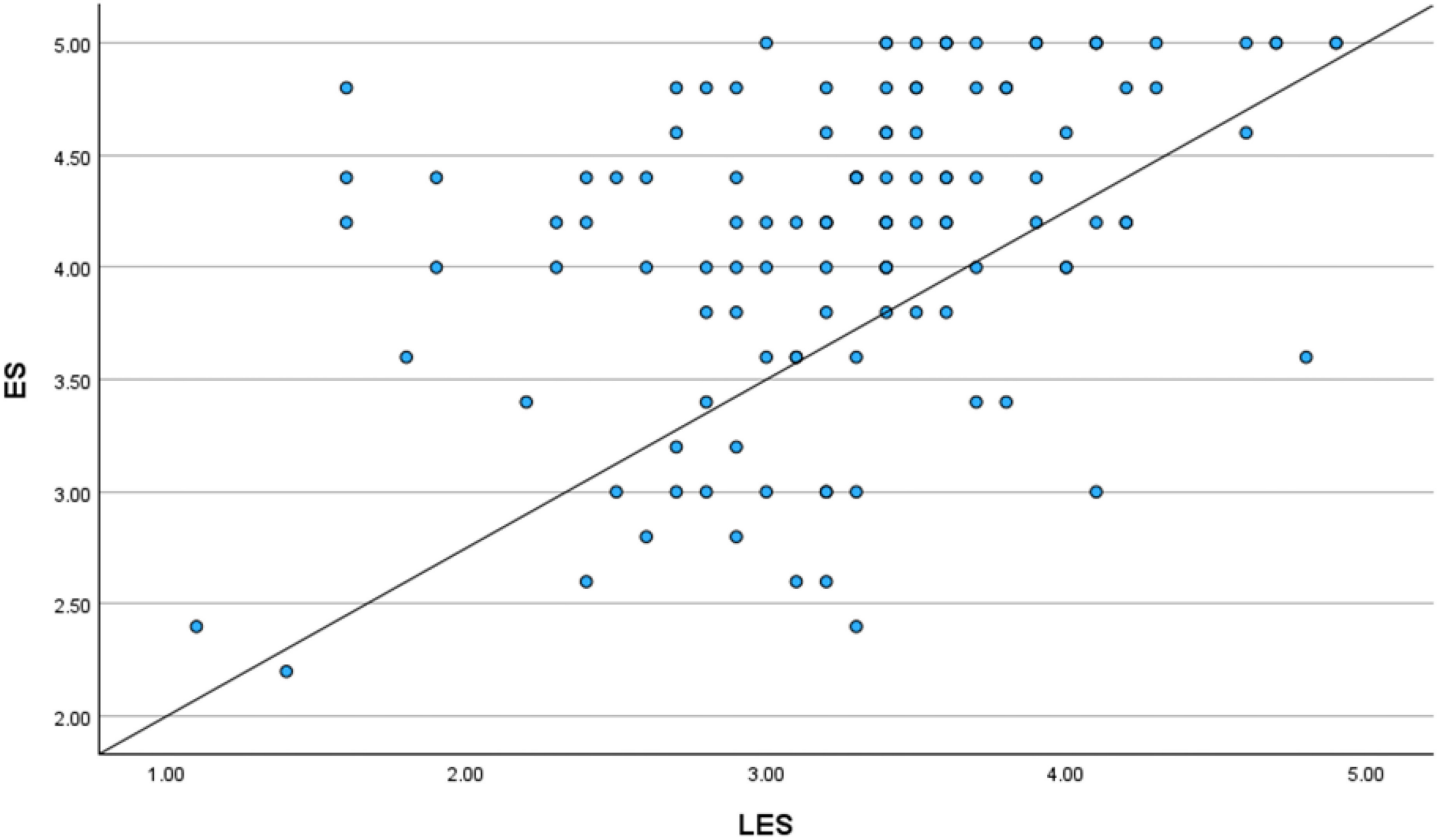
Scatter plot (literary exposure score and empathy score).

**FIGURE 8:**
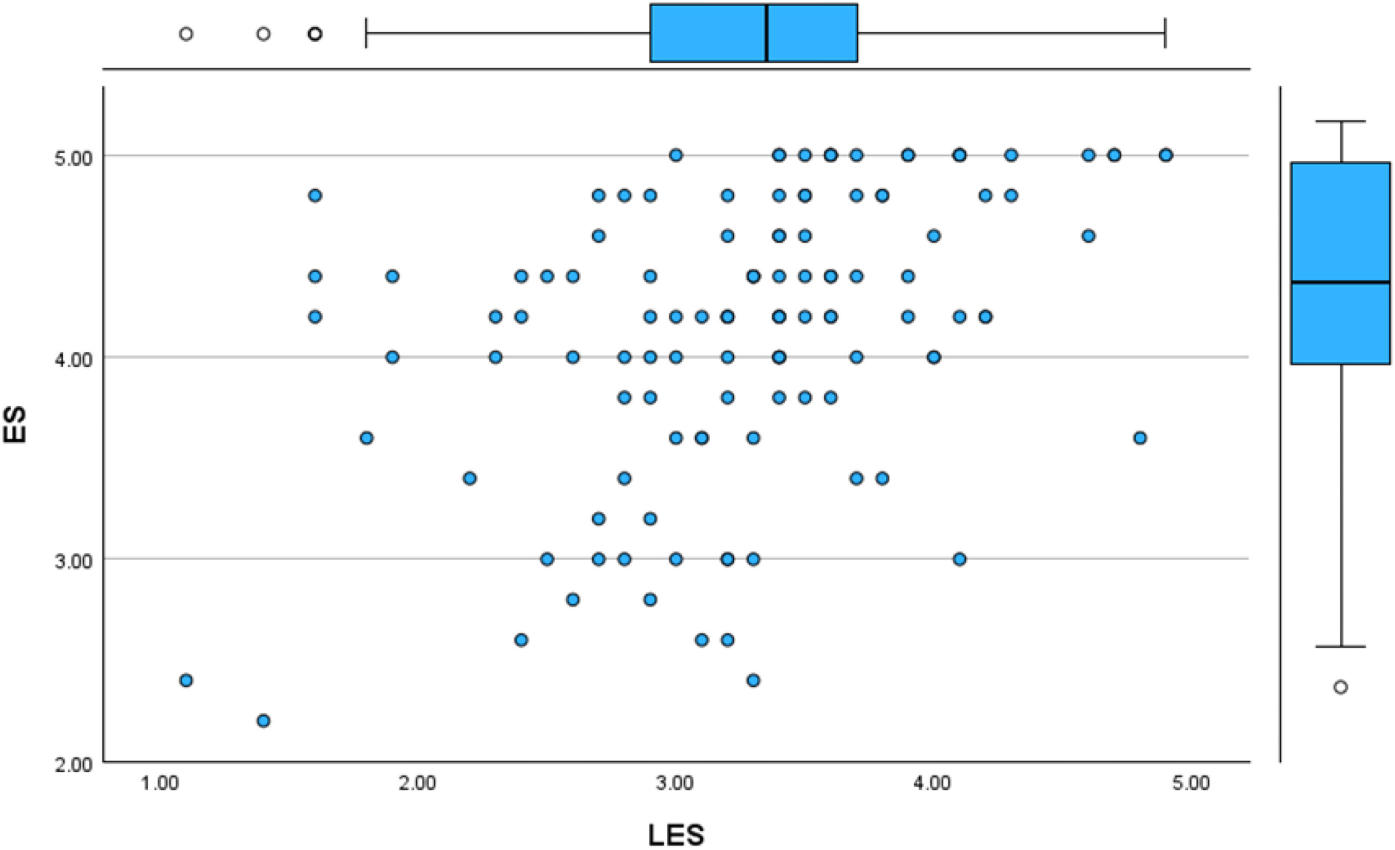
Regression variable plot (literary exposure score and empathy score).

For the first analysis, the focus was on the relationship between LES and ES. The results aligned with the correlational findings, indicating a statistically significant and positive relationship between levels of literary exposure and empathy among medical students and doctors. The scatter plot displayed a positive upward movement (Figures 7, 8). This is in accordance with the correlational analysis that indicates, higher levels of engagement with literature and narratives tended to coincide with higher empathy scores. This pattern highlights the relevance of narrative engagement within clinical contexts, especially with regard to emotional understanding and patient-centered care.

**FIGURE 9:**
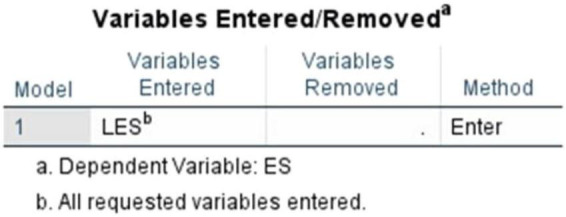
Simple linear regression (literary exposure score and empathy score).

**FIGURE 10:**
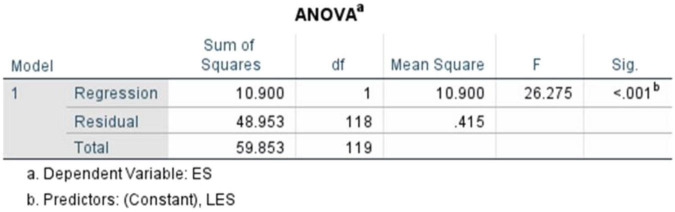
ANOVA table (literary exposure score and empathy score).

The regression model (Figure 9) was observed to be statistically significant, *F*(1,118) = 26.275, *p* < 0.001. The degrees of freedom (1,118) show that Literary Exposure Score was used as the single predictor variable, with 118 representing the remaining observations out of the total 120 responses. As seen in the ANOVA table (Figure 10), the results indicate that the variations in empathy scores are associated with variations in the literary engagement of the participants within the sample. As this analysis is based on cross-sectional data, these findings are interpreted as associative rather than causal.

**FIGURE 11:**
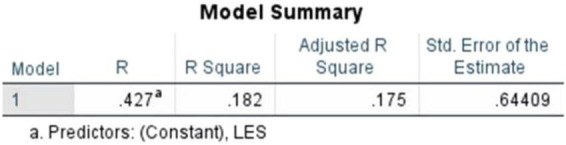
Model summary (literary exposure score and empathy score).

The Model Summary (Figure 11) shows a correlation coefficient (R) of 0.427. The R^2^ value of 0.182 indicates that approximately 18.2% of the variance in empathy scores is associated with literary exposure, within the sample. The adjusted *R*^2^ = 0.175 suggests that this relationship is likely to remain stable across comparable samples. While a substantial proportion of variance in empathy is likely related to other unmeasured factors, the findings indicate that literary exposure is one relevant psychosocial factor within a broad web of influences that shape empathy. The standard error of estimate (0.64409), reflects an acceptable level of dispersion around the regression line. Overall, the results align with the understanding that empathy is shaped by many overlapping influences, including educational, personal and situational, rather than being shaped by one experience alone.

**FIGURE 12:**
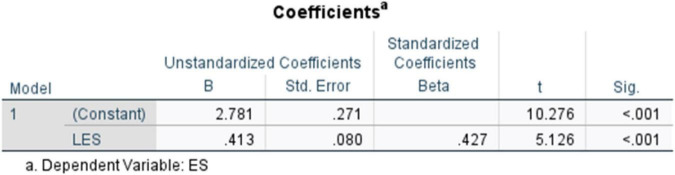
Coefficients table (literary exposure score and empathy score)

Here, the standardized coefficient and the unstandardized coefficient (Figure 12) were recorded at (β = 0.427, *t* = 5.126, *p* < 0.001), (*B* = 0.413, SE = 0.080), respectively. The results indicate a statistically significant positive association between Literary Exposure Score and Empathy Score within the sample.

The findings offer quantitative insight into existing qualitative arguments by Charon (8), Shapiro et al. (10), and Milota et al. (14), which suggest that narrative or literary engagement is associated with empathy and emotional resilience. The present results extend these claims by offering empirical evidence that such engagement is systematically associated with measurable variations in empathy, instead of just reaffirming them. In 2001, Charon claimed that narratives and the very act of storytelling operate as pedagogical tools for nurturing compassionate clinical care. Accordingly, it may be understood as a process that corresponds with observable psychosocial patterns, extending beyond its role as a conceptual framework. In this light, the statistical evidence aligns with and reinforces the conceptual assumption that interpretative engagement with narratives contributes to the development of emotionally responsive clinical practice.

Similarly, Zhao et al. (15) report that regular engagement with stories helped doctors understand emotions better. On the other hand, Kuo et al. (26) showed that narrative medicine training is associated with increased empathy among nursing students. Therefore, this study situates itself alongside the emerging body of work though it does not directly validate the claims. This convergence across studies suggests conceptual consistency and indicates that the relationship between narrative engagement and empathy may be contextually mediated rather than universally uniform.

These results indicate that literary exposure coincides with the inclination toward empathetic and patient-centered care, rather than indicating a direct causal pathway. Together, these findings contribute to strengthening the concept of Narrative Medicine by suggesting that sustained engagement with stories is meaningfully related to deeper emotional awareness in clinical contexts. While this also remains embedded within a broader network of other individual factors.

#### 5.4.2 LES and RS

**FIGURE 13:**
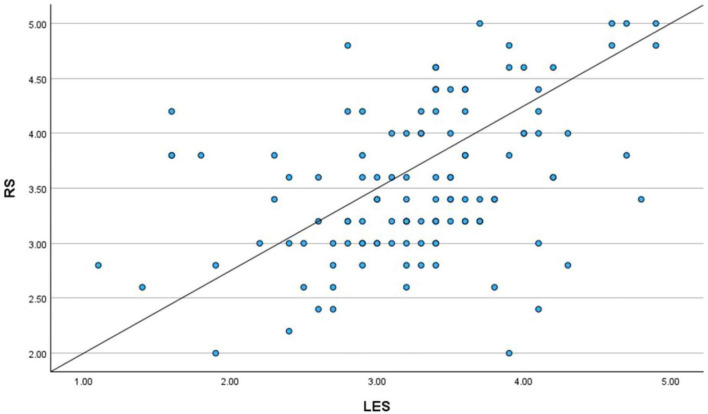
Scatter plot (literary exposure score and resilience score).

**FIGURE 14:**
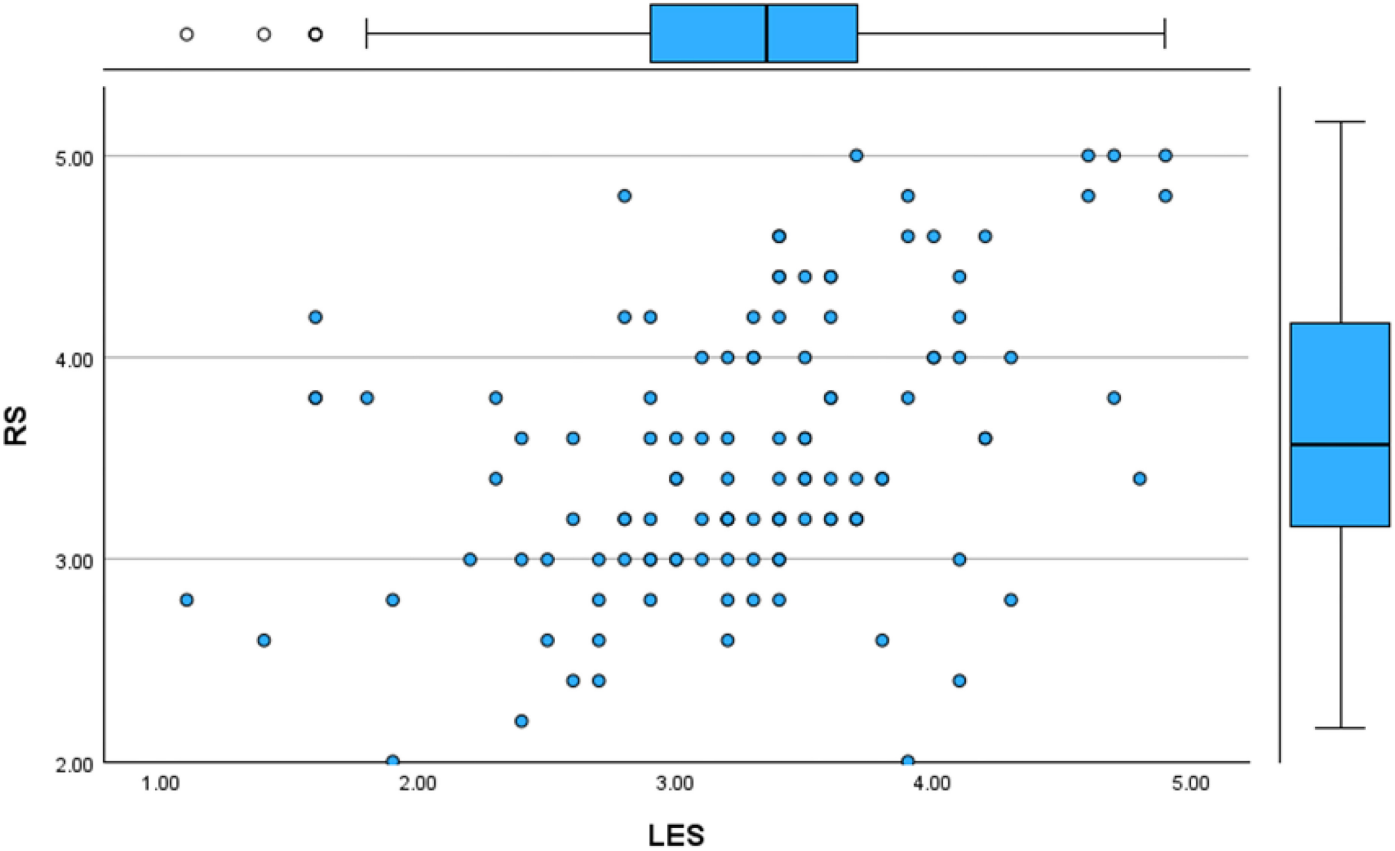
Regression variable plot (literary exposure score and resilience score).

**FIGURE 15:**
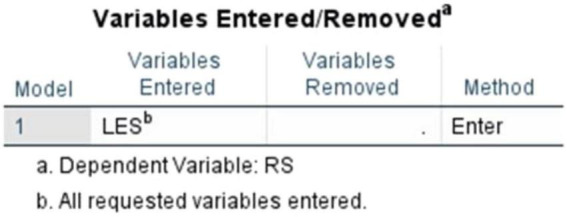
Simple linear regression (literary exposure score and resilience score).

Yet another Simple Regression Analysis was conducted between the Literary Exposure Score (LES) and the Resilience Score (RS) to further examine the observed relationship. The results aligned with the correlation findings and it showed a statistically significant positive connection between the two variables. The scatter plot demonstrated an upward slope, visually suggesting that higher levels of engagement with literature and stories corresponded with higher resilience scores within the sample (Figures 13, 14). These findings (Figure 15) indicate that engagement with narratives is meaningfully related to emotional resilience among medical students and doctors, particularly in the case of emotional and professional struggles.

**FIGURE 16:**
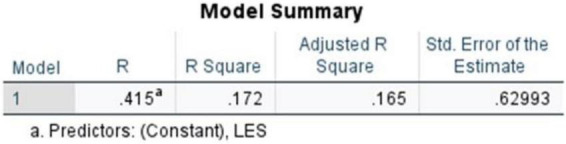
Simple linear regression (literary exposure score and resilience score)

The model summary (Figure 16) demonstrated a moderate positive relationship with an R value of 0.415. The R^2^ value of 0.172 indicates that approximately 17.2% of the variance in resilience scores is associated with literary exposure score within the sample, reflecting a modest but meaningful relationship. The adjusted R^2^ = 0.165 suggests a comparable level of association when accounting for sample size. The standard error of estimate (0.62993) further shows an acceptable level of dispersion around the regression line in the data.

**FIGURE 17:**
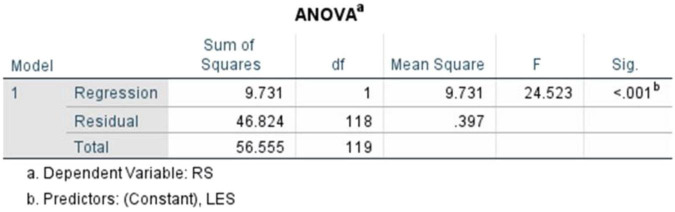
ANOVA table (literary exposure score and resilience score).

The ANOVA table (Figure 17) highlights that the regression model is significant, *F*(1,118) = 24.523, *p* < 0.001, demonstrating a meaningful relationship between literary exposure and resilience within the sample. The degrees of freedom (1,118) indicate that Literary Exposure Score was used as the single predictor variable, with 118 representing the remaining observations out of the total 120 responses. As shown in the table, the results indicate that variations in resilience scores are associated with variations in literary exposure.

**FIGURE 18:**
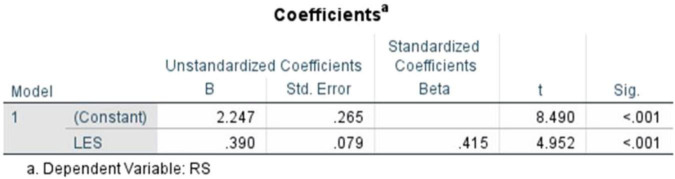
Coefficients table (literary exposure score and resilience score).

The coefficients table (Figure 18) illustrates that the standardized coefficient and the unstandardized constant were recorded at (β = 0.415, *t* = 4.952, *p* < 0.001), *B* = 2.247 (SE = 0.265), respectively. The standardized coefficient reflects a statistically significant and positive association between literary exposure score and resilience score, within the sample. Overall, the findings indicate that higher levels of engagement with literature and narratives are associated with higher levels of emotional resilience among medical students and doctors. The observed pattern suggests that literary engagement is one of the several factors associated with emotional resilience in clinical contexts.

Previous research in the field of medical humanities highlights the relevance of reflective and narrative practices in relation to coping mechanisms in clinical settings (3, 4). Resilience is conceptualized as a trainable quality that is influenced by exposure to emotionally challenging experiences, according to Houpy et al. (3). Similarly, Stoffel and Cain (6) contended that reflective practices, including literary engagement, function as emotional processing tools that may help medical students navigate burnout and emotional exhaustion. The positive associations between Humanities based therapies and markers of coping and well-being are further reported in the works of Shorbagi et al. (22) and Chye et al. (13). Collectively, these perspectives suggest that narrative engagement may operate as a mediating process through which emotional experiences are interpreted, organized and integrated.

When the students and professionals engage with complex narratives, they come across moral ambiguity, emotional vulnerability and endurance. Such encounters can be seen as structured exposures to affectively complex scenarios. This allows individuals to rehearse interpretative and emotional responses in a non-clinical setting. These experiences are often discussed in literature as aspects that are relevant in fostering emotional resilience, by enabling individuals to engage with difficulty in a reflective manner. In this sense, quantitative patterns observed in this study are consistent with the conceptual perspectives of Narrative Medicine, suggesting a meaningful relationship between literary exposure and emotional resilience. It also suggests that this relationship is shaped by broader contextual influences.

The regression results, in alignment with the correlational findings, suggest that literary exposure is positively related to empathy and resilience within the sample. While the statistical model identifies literary exposure as a significant variable in analytical terms, these findings are interpreted as associative rather than causal. Exposure to complex moral and emotional situations in literature may provide contexts in which students encounter themes such as endurance and ambiguity. These engagements may contribute to the development of emotional resilience, a quality that has been discussed in the literature as relevant to patient-care (1, 2). Together, the results suggest that literary engagement is a part of the larger group of educational and experiential factors linked to empathy and resilience, rather than functioning as a singular or deterministic influence.

## 6 Discussion and thematic interpretation

The findings of this research provide empirical support for the key argument of Narrative Medicine, that continuous interaction with stories is meaningfully related to the doctors’ empathy and emotional resilience. The above-mentioned statistical tests indicate a moderate and statistically significant positive association between literary exposure and the characteristic traits under consideration within the regression models. However, these findings are interpreted as associative rather than causal, given the cross-sectional design of this study. Accordingly, both the null hypotheses (H01 and H02) were rejected and the alternative hypotheses (H11 and H12) were affirmed. At the same time, this pattern suggests probability rather than a deterministic relationship. Here, literary exposure operates as a contributing influence within the broader psychosocial and educational framework.

The correlational analyses were conducted between literary exposure and empathy (*r* = 0.427; ρ = 0.456; *p* < 0.001) and between literary exposure and resilience (*r* = 0.415; ρ = 0.412; *p* < 0.001). These results, along with the regression findings, suggest that engaging with stories is systematically associated with emotional and moral dimensions of medical care, while acknowledging that such traits are shaped by educational and contextual influences. The regression coefficient (β = 0.427, *p* < 0.001) indicates a robust positive association between literary exposure and empathy within the sample. Rather than considering empathy as an innate trait, the results align with the conceptual perspectives that position empathy as a dynamic and cultivable ability shaped through interpretative engagement.

When doctors read literature or watch films and series, they come across the complex nature of pain, uncertainty, and human vulnerability. From an analytical perspective, such encounters can be seen as structured forms of reproduced experiences that allow the doctors to engage with affective complexity without clinical pressure. Within the Narrative Medicine scholarship, such encounters help the doctors to see their patients’ experiences as stories, not just as a list of symptoms. Zhao et al. (15) and Liao (1) call this “narrative literacy,” an interpretative skill that sharpens clinical judgment and moral awareness. In this context, the present findings are congruent with the concept that sustained engagement with stories is linked to higher empathy scores, while acknowledging that this relationship is mediated by other cognitive and educational processes rather than functioning in isolation.

Similar to empathy, the results also show a positive association between literary exposure and resilience (β = 0.415, *p* < 01.001). In this study, resilience refers to a doctor’s ability to stay emotionally strong, even when facing difficult or distressing situations. The statistical patterns correspond with existing scholarship in Medical Humanities. Houpy et al. (3) and Stoffel and Cain (4) have argued that reflective practices, such as reading and discussing literature, are linked to the development of medical students’ emotional adaptability. Similarly, Thomas et al. (27) explains that stories build “reflective resilience,” the ability to stay compassionate without feeling drained. This view is also supported by Chang et al. (11). This suggests that narrative engagement may function as a reflective cushion, that helps the individuals process emotional experiences cognitively, not reactively.

Within this interpretative framework, the present findings align with the view that sustained exposure to narratives is associated with higher resilience scores, while acknowledging that resilience is shaped by diverse experiential and institutional factors. When combined, the findings position Narrative Medicine at the intersection of emotional and cognitive aspects of medical practice. Here, literary engagement is associated with both empathy and resilience. The results suggest that these two qualities may operate in relation to one another rather than as isolated traits. This interrelationship points toward an integrated model of clinical competence, where emotional understanding and endurance are co-produced.

The observed relationships are moderate in strength indicating that literary exposure accounts for a limited proportion of variance in empathy and resilience. Therefore, these findings should be interpreted as indicative rather than explanatory. Various other factors such as personal, educational and social factors influence these psychosocial traits. Empathy, in this case, reflects the capacity to understand their patients’ experiences, while resilience provides them the strength to sustain that understanding without emotional depletion. The statistical patterns observed in this study indicate that higher levels of literary exposure correspond with higher scores on both traits. Conceptually, this association supports the view that empathetic sensitivity and emotional resilience function together within clinical settings. However, neither of these can be reduced to a single influencing factor.

A substantial proportion of variance in empathy and resilience is unexplained within the present model, indicating the likely influence of unmeasured confounding variables. Factors such as personality traits, educational environment, prior clinical exposure and socio-cultural background may significantly contribute to these psychosocial constructs. The absence of these variables suggests that literary exposure should be understood as one contributing factor within a broader, multifactorial developmental framework.

Even moderate relationships are meaningful in psychosocial research as the complex traits such as empathy and resilience emerge from multiple other influences. So, literary exposure and engagement may be understood as one of the contributing factors in a wide context. Beyond this, the implications of this study extend into the ethical realm of clinical practice. Literary engagement may help sensitize practitioners to the lived realities of patients before clinical encounters occur. This concern is central to the debates on medical ethics. In this case, “iatrogenic suffering,” a term used by Kuhl (5) to describe the form of pain caused through clinical detachment and communicative failure becomes important.

The positive associations identified in this study resonate with Rita Charon’s (8) argument that listening to patients’ stories is a moral activity that prevents depersonalization. Instead of placing the present findings as evidence of direct prevention, they offer quantitative support to the existing qualitative accounts within Narrative Medicine. Humanities-based practices such as reading and reflective writing may contribute to more reflective and compassionate clinical practice, as suggested by Rafaqat et al. (9) and Shorbagi et al. (22). The results of this study align with these perspectives suggesting that literary engagement coexists with capacities that are frequently discussed in relation to ethical clinical practice.

Empathy and resilience are often termed as “soft skills,” however within empirical research they emerge as measurable and analytically relevant constructs. Integrating literary studies into medical curricula may offer a flexible educational approach for engaging with these psychosocial traits. The conventional biomedical structure prioritizes technical proficiency and the humanities-based approaches foreground the experiential and interpretative aspects of patient care. The statistical analyses conducted in this study identified a significant positive relationship between narrative exposure and both empathy and resilience. These findings correspond with contemporary debates around narrative training in medical education (1, 13, 27), while remaining interpretative and not causal in scope. Yet, the findings should be understood as context-bound and indicative of patterns rather than definitive explanatory models.

From a practical and educational perspective, the observed associations suggest that literary engagement may function as a complementary pedagogical resource and not as a primary intervention. Even modest differences in empathy and resilience are meaningful within clinical training contexts, where cumulative improvements in emotional awareness and reflective capacity can influence patient communication, ethical sensitivity and professional well-being over time. These findings suggest that narrative-based approaches may be considered as part of a broader, interdisciplinary medical education framework.

In conclusion, the results indicate that literature and medicine are not separate domains but intersect meaningfully within medical education. Both the null hypotheses were rejected based on the empirical findings. These traits appear to be interrelated within the narrative contexts examined in this study. This suggests that literary engagement may contribute to a more integrated form of clinical awareness without functioning as a singular or determining factor.

## 7 Conclusion

This study suggests that reading literature and engaging with stories may have relevance beyond leisure within medical education. The findings suggest that literary engagement may be meaningfully situated within measurable psychosocial dimensions of medical training. The results indicate statistically significant and moderately positive correlations between Literary Exposure and Empathy (*r* = 0.427; ρ = 0.456; *p* < 0.001) and Resilience (*r* = 0.415; ρ = 0.412; *p* < 0.001). Furthermore, the linear regression analysis, which aligns with the correlational findings illustrates the idea that literary exposure, as one of the contributing factors within the statistical models, is associative rather than causal in nature.

These patterns align with the existing arguments in Narrative Medicine, especially in the works of Rita Charon, who conceptualizes storytelling as central to compassionate clinical practice. Within this interpretative framework, engagement with stories corresponds with an inclination to recognize patients as individuals immersed in emotional and experiential narratives rather than abstract clinical traits.

Narrative Medicine proposes that reading and interpreting stories may contribute to doctors’ personal and professional development. Through this process, doctors also develop the practice of reflection, which helps them to stay emotionally stable while caring for others. The results of this study suggest that empathy and resilience may be interconnected within the narrative contexts examined. In this sense, literature may be understood as a reflective space in which the complexities of lived experiences are represented and interpreted. Engagement with such narratives is associated with emotional and ethical dimensions of medical practice.

The findings suggest the relevance of the humanities, especially literature, within medical education. In this study, higher levels of literary engagement were associated with empathy and resilience, qualities that are often linked to compassionate and reflective clinical practice. Rather than presenting these results as definitive evidence of pedagogical impact, they provide empirical support for existing arguments that humanities-based approaches such as reading, engagement with narrative media, and reflective writing are associated with the psychosocial traits examined in this study. The present findings align with these perspectives suggesting that narrative exposure corresponds with forms of empathetic sensitivities and emotional resilience. Therefore, this study supports the consideration of integrating literary narratives in medical education, while recognizing that their influence on psychosocial traits remains correlational rather than causal.

## 8 Limitations, scope, and future directions

This study examined the relationship between literary exposure and selected psychosocial dimensions relevant to medical education. It brings the ideas of Narrative Medicine into dialogue with empirical data. The findings identify statistically significant and moderately positive associations between literary exposure, empathy, and resilience, contributing empirical support to ongoing discussions about the role of the humanities in medical education. Within the examined sample, the observed patterns indicate that narrative engagement corresponds with certain characteristic traits such as empathy and resilience that are often linked to compassionate clinical practice. This study contributes to the literature by examining these associations in a non-Western context; however, the findings should be interpreted within the limits of the present sample and setting.

However, this is a cross-sectional study, therefore does not allow for causal inferences or the examination of changes over time. This study acknowledges the possibility of self-selection bias. For instance, the individuals who are already more empathetic may be more inclined toward literary engagement, or other unmeasured factors shape both reading practices and psychosocial traits. These alternative explanations are acknowledged and considered in the discussion. The use of non-probability (purposive and convenience) sampling limits the generalizability of the findings, and future studies may benefit from larger, more representative samples across institutional contexts.

The study also relies on self-reported measures of empathy and resilience, which may be subject to response bias, including social desirability and subjective interpretation of questionnaire items. As a result, participants’ responses may not fully reflect their actual clinical behavior or emotional competencies. Additionally, the absence of a control or intervention group limits the ability to compare outcomes or attribute observed differences specifically to literary exposure. Besides, the possibility of evaluation bias cannot be fully excluded. Participants may have responded in ways influenced by their awareness of socially desirable attributes such as empathy and resilience, or by their familiarity with academic and clinical expectations within their training environments.

Future research may use mixed-method or longitudinal approaches to better understand long-term patterns and possible developmental pathways. Factors such as burnout, ethical stress, and communication challenges could expand the scope of this work and provide a more complete view of how narrative engagement relates to medical practice. Future research may also benefit from incorporating behavioral or observational measures to complement self-reported data.

## Data Availability

The original contributions presented in the study are included in the article/supplementary material, further inquiries can be directed to the corresponding author.
